# Aluminosilicate-Supported Catalysts for the Synthesis of Cyclic Carbonates by Reaction of CO_2_ with the Corresponding Epoxides

**DOI:** 10.3390/molecules27248883

**Published:** 2022-12-14

**Authors:** Luciano Atzori, Adrien Comès, Luca Fusaro, Carmela Aprile, Maria Giorgia Cutrufello

**Affiliations:** 1 Dipartimento di Scienze Chimiche e Geologiche, Università di Cagliari, 09042 Monserrato, Italy; 2 Unit of Nanomaterial Chemistry, Department of Chemistry, University of Namur, 5000 Namur, Belgium; 3 Consorzio Interuniversitario Nazionale per la Scienza e Tecnologia dei Materiali (INSTM), Unità di Cagliari, 50121 Firenze, Italy

**Keywords:** cyclic carbonates synthesis, CO_2_ conversion, supported catalysts, Al_SBA-15, imidazolium chloride

## Abstract

Functionalized aluminosilicate materials were studied as catalysts for the conversion of different cyclic carbonates to the corresponding epoxides by the addition of CO_2_. Aluminum was incorporated in the mesostructured SBA-15 silica network. Thereafter, functionalization with imidazolium chloride or magnesium oxide was performed on the Al_SBA-15 supports. The isomorphic substitution of Si with Al and the resulting acidity of the supports were investigated via ^27^Al magic angle spinning (MAS) nuclear magnetic resonance (NMR) spectroscopy and NH_3_ adsorption microcalorimetry. The Al content and the amount of MgO were quantified via inductively coupled plasma optical emission spectroscopy (ICP-OES) analysis. The anchoring of the imidazolium salt was assessed by ^29^Si and ^13^C MAS NMR spectroscopy and quantified by combustion chemical analysis. Textural and structural properties of supports and catalysts were studied by N_2_ physisorption and X-ray diffraction (XRD). The functionalized systems were then tested as catalysts for the conversion of CO_2_ and epoxides to cyclic carbonates in a batch reactor at 100 or 125 °C, with an initial CO_2_ pressure (at room temperature) of 25 bar. Whereas the activity of the MgO/*x*Al_SBA-15 systems was moderate for the conversion of glycidol to the corresponding cyclic carbonate, the Al_SBA-15-supported imidazolium chloride catalysts gave excellent results over different epoxides (conversion of glycidol, epichlorohydrin, and styrene oxide up to 89%, 78%, and 18%, respectively). Reusability tests were also performed. Even when some deactivation from one run to the other was observed, a comparison with the literature showed the Al-containing imidazolium systems to be promising catalysts. The fully heterogeneous nature of the present catalysts, where the inorganic support on which the imidazolium species are immobilized also contains the Lewis acid sites, gives them a further advantage with respect to most of the catalytic systems reported in the literature so far.

## 1. Introduction

**Scheme 1:**
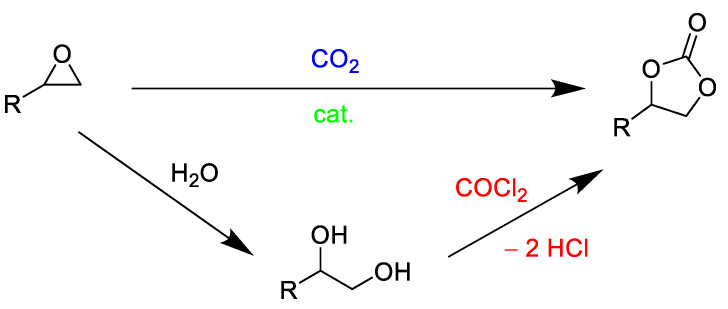
CO_2_ conversion into cyclic carbonates.

The synthesis of cyclic carbonates through the reaction of CO_2_ with epoxides represents a significant opportunity to transform waste into valuable chemicals while avoiding the use of phosgene (Scheme 1). This reaction is at the basis of industrial production, through which about 100 kt of cyclic carbonates are obtained per year; such value is also expected to increase, as the market of chemicals produced from cyclic carbonates (solvents, paint strippers, lithium batteries, biodegradable packaging, etc.) is constantly expanding [1,2,3].

The industrial process is carried out using mainly nucleophile catalysts, which favor the opening of the epoxy ring and, subsequently, the insertion of carbon dioxide. The classic industrial catalysts are mainly ammonium or phosphonium salts, which require high temperatures and pressures. In addition, they are generally difficult to recover. For these reasons, the future challenge should be focused on finding active solid materials able to promote CO_2_ conversion in milder conditions with easily recoverable and reusable catalysts [1,4].

Both homogeneous and heterogeneous catalysts have been studied for the cycloaddition of CO_2_ to epoxides. Numerous homogeneous systems, such as phosphines [5,6], organic bases [7,8], organometallic complexes [9,10,11], and ionic liquids (ILs) [12,13,14,15,16,17], have been proposed. However, though showing a high catalytic activity, they suffer from several disadvantages, related to their degradation, separation processes, and, consequently, reusability. In this regard, solid catalysts have to be considered preferable, even though large efforts have to be conducted in order to increase their activity [1,18].

**Scheme 2:**
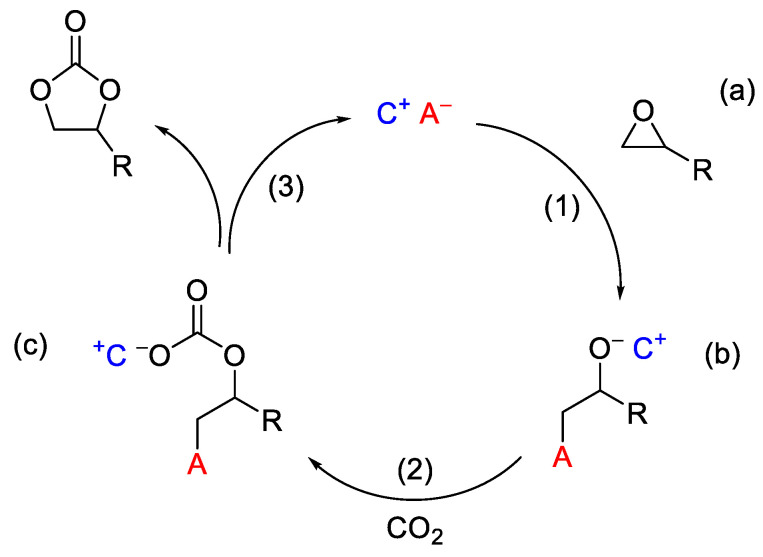
Reaction mechanism for CO_2_ cycloaddition to epoxide with ionic liquids (C^+^ A^−^).

Different classes of ionic liquids, based on quaternary ammonium [12], phosphonium or pyridinium [19], or imidazolium [20] salts, have shown remarkable catalytic performance for the CO_2_ conversion to organic carbonates. The reaction mechanism at the basis of the catalytic activity of such ILs is depicted in Scheme 2 [21]. In the first step (1), the opening of the epoxy ring (a) occurs as a consequence of the nucleophilic attack by the anion species A^−^, with the resulting intermediate (b) being stabilized by the interaction with the cationic moiety. Next, the insertion of CO_2_ (2) leads to the formation of the (c) species, from which the formation of the five-membered cyclic carbonate occurs as a result of the elimination mechanism, thus restoring the catalytic ionic couple (C^+^ A^−^) (3). From this mechanism the prominent role of the anionic species clearly emerges, which has to be good both as a nucleophile and as leaving group (this is why halides are generally selected to obtain high performances). In addition, the role of the cationic moieties appears also important, being responsible for the stabilization of the (c) and (b) intermediates. In this regard, the presence of suitable Lewis acid species has been indicated as beneficial, due to the coordination of the oxygen at the three-membered ring, which can favor the nucleophilic attack and then the ring opening of the epoxide [22,23].

Since the reusability of the ILs represents the main drawback, their immobilization on suitable solid materials has attracted increasing interest [2,18,21,24]. Different catalysts have been prepared by anchoring imidazolium-based salts on polymer matrices [25,26,27,28], with very interesting results obtained by using biopolymers, such as chitosan, which appears as a very promising support thanks to the presence of several hydroxyl groups able to stabilize the (b) and (c) intermediates shown in Scheme 2 [29,30]. The use of 3-*n*-butyl-1-propyl-imidazolium grafted on SiO_2_ has been reported by Xiao et al. in [31], where the influence of different metal salts acting as co-catalysts was also studied. The results indicated that high propylene carbonate yields are obtained by using ZnCl_2_. However, a decrease in activity was observed for recycling tests, as a consequence of the occurrence of leaching phenomena. High catalytic performances have been obtained by Dai et al. [32] on SBA-15 and Al_SBA-15 functionalized with 3-(2-hydroxyl-ethyl)-1-propylimidazolium bromide, though the occurrence of leaching was also observed to some extent up to three recycling tests.

In extension, nitrogen-based organic molecules (e.g., alkyl amines, adenine, guanine) supported on different siliceous materials, such as MCM-41 and SBA-15, have been found to be highly active for converting CO_2_ into cyclic carbonates. Very good results were obtained by using Al_SBA-15 and Ti_SBA-15, since the introduction of Al^3+^ or Ti^4+^ enhances the acidic properties of the catalyst, promoting the epoxides adsorption and activation [7,33,34,35]. Good results have also been displayed by catalysts prepared by anchoring Cr(salen) complexes on amino-functionalized SiO_2_, ITQ-2, and MCM-41 [36,37,38], with the need for solvents or co-catalysts and their poor reusability being the main drawbacks.

Metal oxides with basic properties, such as MgO, represent an interesting option for fully inorganic systems for catalyzing CO_2_ cycloaddition to epoxides, since they are relatively cheap and simple to prepare. Promising results have been obtained by using MgO in the presence of dimethylformamide (DMF), which plays the twofold role of solvent and active species in the reaction mechanism [39]. Yamaguchi et al. [40] have shown that, in the presence of DMF, Mg-Al mixed oxides prepared by using hydrotalcites as precursors also provide interesting performances due to the cooperation of acid and base sites in the epoxy ring opening. Such a synergistic effect has also been confirmed by other studies carried out on Mg-Zn-Al mixed oxides as well as on zeolites modified with alkali metal oxides, in the presence of suitable amounts of water [41,42]. Despite the large number of studies carried out in order to find solid materials able to promote the production of cyclic carbonates, heterogeneous catalysts are significantly less active than homogeneous alternatives. In this regard, the immobilization of organic bases and organometallic complexes on suitable supports can be considered a good strategy to obtain high activity while solving problems related to the separation and reusability of the homogeneous catalysts. However, only a minor part of the literature deals with completely heterogeneous catalysts, in which both the inorganic Lewis acid sites and the immobilized imidazolium species are simultaneously present. Among these, only a few report on the use of Al_SBA-15 as a support [32,43], although a study on the effect of the aluminum content on the catalytic performance is completely missing. Moreover, to the best of the present authors’ knowledge, no studies mentioning the use of Al_SBA-15 in combination with MgO have been reported.

**Scheme 3:**
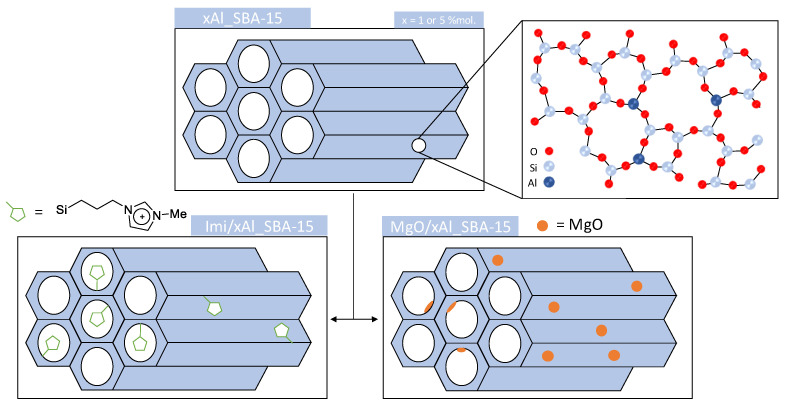
Synthesis of *x*Al_SBA-15 (*x* = 1 or 5) supports and their functionalization by either imidazolium chloride (Imi/*x*Al_SBA-15) or MgO (MgO/*x*Al_SBA-15).

Here, the synthesis of two Al_SBA-15 systems, i.e., mesostructured aluminosilicates with acidic properties, is reported. These materials were thereafter functionalized by either imidazolium chloride (immobilized ionic liquid) or magnesium oxide (basic metal oxide) as depicted in Scheme 3. The initial supports and the final catalysts were characterized by different techniques such as inductively coupled plasma optical emission spectroscopy (ICP-OES), combustion chemical analysis, X-ray diffraction (XRD), N_2_ physisorption, ^27^Al, ^29^Si, and ^13^C magic angle spinning (MAS) nuclear magnetic resonance (NMR) spectroscopy, and NH_3_ adsorption calorimetry. The activity of all catalysts, as well as the influence of the aluminum content, was scoped via the conversion of different epoxides. In addition, recycling tests were performed on selected samples to evaluate the materials’ reusability in multiple catalytic cycles.

## 2. Results and Discussion

### 2.1. Materials Characterization

The initial synthesis of the two *x*Al_SBA-15 (*x* = 1 or 5) systems was performed, where *x* represents the theoretical Al content expressed in mol%. Prior to the functionalization of the aluminosilicates with imidazolium or MgO, the two supports 1Al_SBA-15 and 5Al_SBA-15 were fully characterized, notably by ICP-OES, N_2_ physisorption, low-angle XRD, ^27^Al NMR and NH_3_ adsorption microcalorimetry.

The aluminum content determined by ICP-OES (Table 1) was similar to the nominal value for 1Al_SBA-15, whereas a more significant difference between the experimental and the nominal value was observed for the sample containing the higher aluminum content.

**Figure 1:**
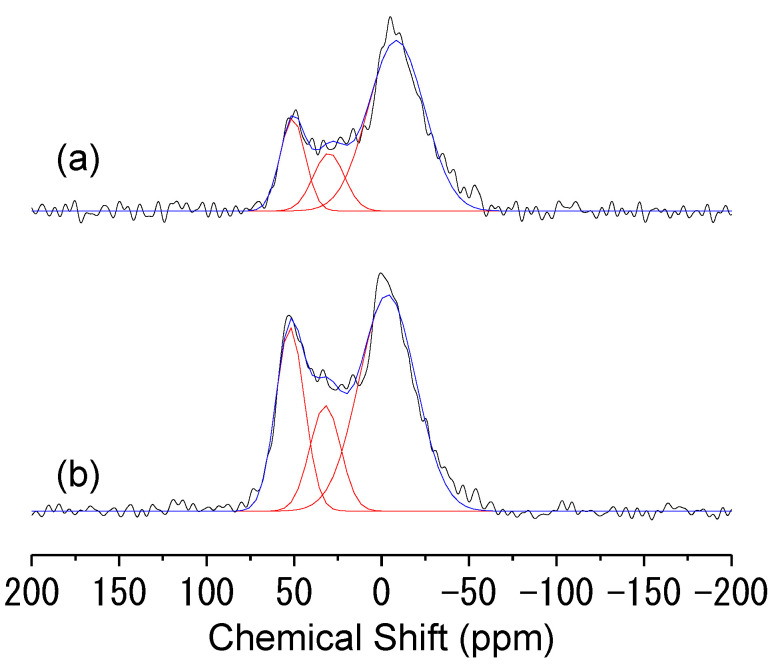
^27^Al MAS NMR spectra of 1Al_SBA-15 (**a**) and 5Al_SBA-15 (**b**).

The insertion of Al in the silica architecture was evaluated via ^27^Al NMR spectroscopy. From the spectra reported in Figure 1, it emerged that only a limited portion of the aluminum present in the supports was incorporated as a single site in the silica framework. Indeed, both ^27^Al NMR spectra are characterized by broad bands resulting from the partial overlapping of different contributions. In both spectra, at least two signals centered at about 53 and 0 ppm are clearly visible. According to the literature, the signal at 53 ppm can be ascribed to the tetrahedrally coordinated framework Al atoms, while the contribution at 0 ppm can be associated with octahedrally coordinated extra-framework Al species [44,45]. Although not clearly visible, an additional contribution at about 30 ppm can be glimpsed, which, according to some studies carried out on dealuminated zeolites, can be reasonably associated with the presence of additional surface five-coordinated Al species [46,47]. Therefore, the percentage of framework aluminum reported in Table 1 was calculated by fitting the NMR profiles with three contributions and taking into account only the contribution at 53 ppm. Although for 5Al_SBA-15 the total amount of aluminum incorporated in the material is lower than the theoretical value, the percentage of framework aluminum is higher than for the other support.

**Figure 2:**
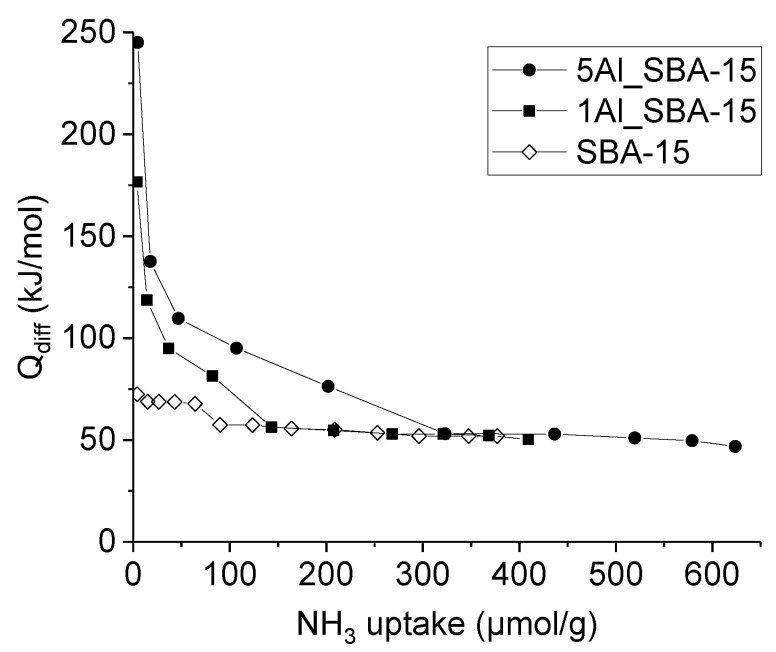
Differential heat of adsorption as a function of NH_3_ uptake at 80 °C for 1Al_SBA-15 and 5Al_SBA-15. For comparison, data for pure silica SBA-15 are also reported.

The insertion of Al in the silica structure is known to generate a combination of Brønsted and Lewis acidity. The acidic properties of the *x*Al_SBA-15 supports were studied by means of NH_3_ adsorption microcalorimetric measurements. In Figure 2, the differential heat of adsorption (*Q*_diff_) is reported as a function of the ammonia uptake for all the prepared supports. The results for an Al-free SBA-15 sample have also been reported for comparison. The *x*Al_SBA-15 samples show acidic properties significantly more pronounced than SBA-15, in terms of both the number and strength of the sites. Whereas the pure silica SBA-15 curve always lies below 70 kJ/mol, the *x*Al_SBA-15 samples show very high (>150 kJ/mol) initial *Q*_diff_ values which then decrease as the NH_3_ uptake increases, thus indicating a high heterogeneity of the adsorbing sites, until reaching a rather constant value (50–58 kJ/mol). Indeed, such a set (260–290 μmol/g) of homogeneous weak acid sites appears to be practically the same present in SBA-15. In agreement with previous studies on pure silica samples, in order to disregard the non-specific and/or physical adsorption occurring on SBA-15, the total concentration of acid sites can be obtained by neglecting the fraction of ammonia uptake corresponding to differential heats below 70 kJ/mol [48,49]. As expected, the acid site concentration increases with the Al content, resulting to be ca. 110 and 235 μmol/g for 1Al_SBA-15 and 5Al_SBA-15, respectively.

**Figure 3:**
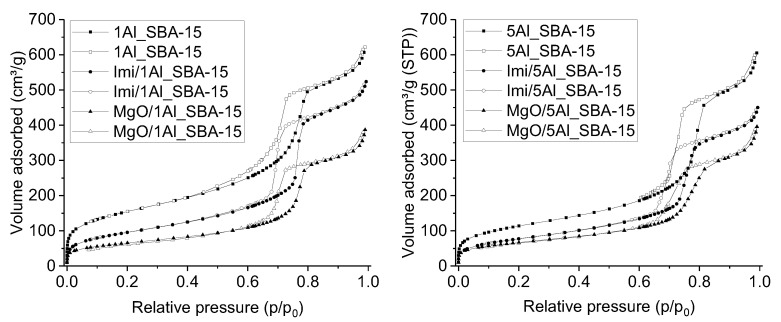
N_2_ adsorption (full symbols) and desorption (open symbols) isotherms for the *x*Al_SBA-15 supports and the Imi/*x*Al_SBA-15 and MgO/*x*Al_SBA-15 catalysts.

The N_2_ adsorption/desorption isotherms of the supports are reported in Figure 3. The isotherms can be classified as type IVa, with an H1 hysteresis loop at high relative pressures, typical for mesoporous materials [50]. The pore size distribution (PSD) curves (Figure S1), obtained by applying the BJH method to the desorption branch of the isotherms, appeared quite narrow and centered at around 7 nm. The specific BET surface area (*S*_BET_) and the specific pore volume (*V*_p_) obtained from the N_2_ adsorption data are reported in Table 1.

Although high, the *S*_BET_ and *V*_p_ values of the *x*Al_SBA-15 supports are definitely lower than those typically obtained for pure silica SBA-15 materials (usually in the ranges 800–1100 m^2^/g and 1.20–1.90 cm^3^/g, respectively) [51,52,53,54]. The detrimental effect of the aluminum content on the surface area was already observed for similar samples [48] and can be ascribed to the presence of aluminum extraframework species.

**Figure 4:**
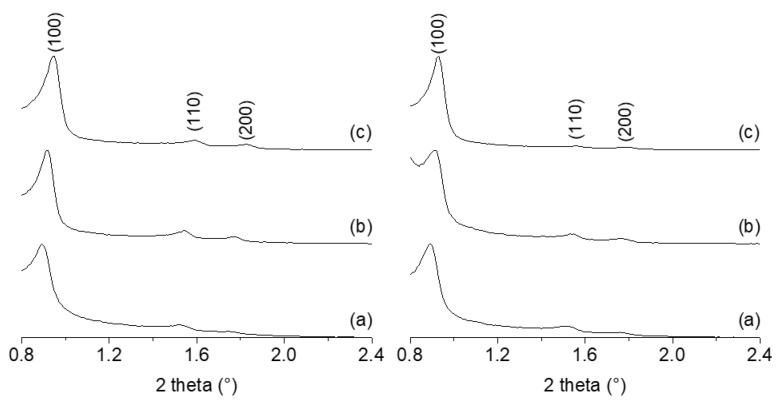
Low-angle X-ray diffraction patterns of *x*Al_SBA-15 (**a**), Imi/*x*Al_SBA-15 (**b**), and MgO/*x*Al_SBA-15 (**c**). **Left**: *x* = 1; **right**: *x* = 5.

In Figure 4, the low-angle X-ray diffraction patterns of the *x*Al_SBA-15 supports are reported. As expected, the samples show three well-resolved peaks which can be indexed as the (100), (110), and (200) reflections associated with a hexagonal symmetric pore structure.

Both solids were employed as supports for subsequent functionalization. On the one hand, the silica surface was decorated with imidazolium chloride via the method using a one-pot grafting with 3-(chloropropyl)-trimethoxysilane and N-methylimidazole [55,56]. The resulting solids were denominated as Imi/*x*Al_SBA-15. On the other hand, both *x*Al_SBA-15 were impregnated with magnesium nitrate using a two-solvent method [57,58]. The materials were then calcined at 450 °C to generate MgO in the porous structure. The resulting materials were denominated as MgO/*x*Al_SBA-15. The experimental details for both procedures are reported in Section 3.

The imidazolium content in the Imi/*x*Al_SBA-15 catalysts was evaluated from the nitrogen content obtained by the combustion chemical analysis. The Al, Si, and Mg content in the MgO/*x*Al_SBA-15 catalysts was determined through ICP-OES. The results of these analyses are reported in Table 1. The amount of imidazolium retained by the two supports results to be affected by the Al content. Most likely, the increase in the number of acid sites with aluminum content, as revealed by adsorption microcalorimetry, in turn, enhances the tendency of the support to interact with the imidazolium function.

**Figure 5:**
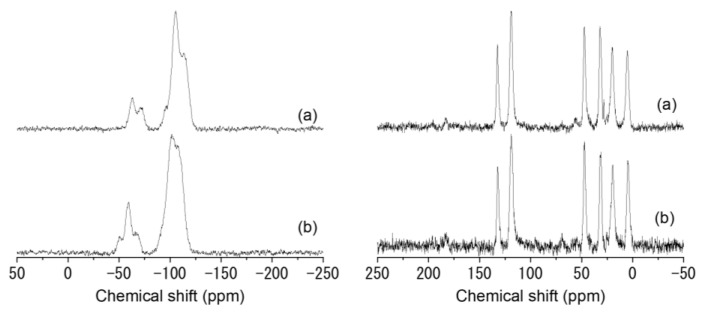
^29^Si (**left**) and ^13^C (**right**) CP-MAS NMR spectra of Imi/1Al_SBA-15 (**a**) and Imi/5Al_SBA-15 (**b**).

Solid-state NMR spectroscopy was employed to assess the correct anchoring of imidazolium species to the silica surface. In Figure 5 (left), the ^29^Si cross-polarization magic-angle spinning (CP-MAS) NMR spectra of the Imi/*x*Al_SBA-15 catalysts are reported. In both spectra, two groups of signals ascribed to Q and T species can be distinguished. The presence of signals at −75, −60, and −50 ppm which can be assigned to -CH_2_-Si(OSi)_3_ (T^3^), -CH_2_-Si(OSi)_2_(OH) (T^2^), and -CH_2_-Si(OH)_2_(OSi) (T^1^) contributions, respectively, constitute the proof of the covalent anchoring of the organic functionalities. The signals in the region between −125 and −85 ppm can be deconvoluted into three different contributions centered at ca. −115, −105, and −95 ppm. corresponding to Si(OSi)_4_ (Q^4^), Si(OSi)_3_OH (Q^3^), and Si(OSi)_2_(OH)_2_ (Q^2^) species.

The ^13^C CP-MAS NMR spectra of the two materials reported in Figure 5 (right) demonstrate the presence of the imidazolium salt. The two characteristic peaks around 125 ppm can be assigned to the aromatic carbon from the imidazolium ring while the peaks at 10, 20, and 45 ppm are, respectively, assigned to Si-CH_2_-CH_2_-CH_2_-N from the propyl chain. The peak at 30 ppm can be attributed to the N-methyl group [55,56,59]. The absence of any other peak (particularly CH_2_-Cl) indicates the complete conversion of the chloropropylsilane to N-methylimidazolium.

At variance with what was observed for the imidazolium function, the amount of MgO incorporated in the two materials (Table 1) was not affected by the different aluminum content in the supports.

The wide-angle XRD was employed to evaluate the incorporation of MgO in the pores. No diffraction peaks (Figure S2) were detected, suggesting the presence of only nanosized crystals. The very large signal centered at 2 theta = 23° is typical of the amorphous SiO_2_ structure.

All materials were submitted to N_2_ physisorption analysis (Figure 3) and low-angle XRD (Figure 4) to evaluate the textural and structural modification compared to the initial supports. Both specific surface area and pore volume values of all the catalysts were lower than those of the corresponding supports (Table 1), as a consequence of the functionalization procedure. The analysis of the X-ray diffraction pattern highlights that this decrease does not affect the regular arrangement of the pores. 

Considering all the favorable textural and structural properties of the solids together with the corresponding imidazolium or MgO functionalization, the materials were employed as catalysts for the conversion of CO_2_ and epoxides to cyclic carbonates. Glycidol was initially selected as the reactant. According to what was reported on Mg-Al mixed oxide catalysts by Yamaguchi et al. [40], the presence of only *x*Al_SBA-15 would not lead to any conversion. Their experiment using a physical mixture of Al_2_O_3_ and MgO clearly demonstrated the importance of the proximity of Mg and Al sites to have a cooperative action.

### 2.2. Catalytic Results

#### 2.2.1. CO_2_ Addition to Glycidol

**Figure 6:**
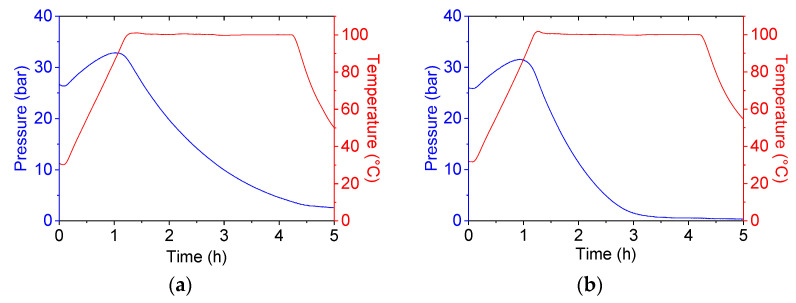
Pressure profile during the glycidol conversion to the corresponding cyclic carbonate for Imi/1Al_SBA-15 (**a**) and Imi/5Al_SBA-15 (**b**). They, respectively, refer to Entries 3 and 4 in Table 2.

The catalytic tests performed in the presence of MgO/*x*Al_SBA-15 catalysts displayed a moderate activity for glycidol conversion which increases with the enhanced Al content (Table 2; Entries 1 and 2). The epoxide conversion was more efficient when the corresponding imidazolium-containing materials were employed (Entry 1 vs. 3 and Entry 2 vs. 4). This first series of catalytic experiments highlighted the superior activity of the imidazolium-based catalysts, thus indicating that the presence of a nucleophile (as catalytically active species) drastically overpasses the effect of possible acid/base synergy at the catalyst surface. It deserves to be mentioned that the substoichiometric amount of CO_2_ fed in the reactor probably affected the glycidol conversion, in particular for the Imi/*x*Al_SBA-15 catalysts. The decrease in pressure directly correlated to the CO_2_ consumption reported in Figure 6 demonstrates that a total (or nearly total) consumption of CO_2_ can be claimed, which is not the case for MgO/5Al_SBA-15 nor MgO/1Al_SBA-15 with, respectively, 22 bar and 27 bar of pressure remaining—at 100 °C—after 3 h of reaction. The evolution of the pressure is also directly correlated to the conversion. It is important to underline the positive influence of the higher amount of Al. For MgO/*x*Al_SBA-15 catalysts (Entry 1 vs. 2), which have very similar MgO content (*cf*. Table 1), such effects can be reasonably ascribed to aluminum acting as a co-catalyst for the ring-opening of the epoxy-ring. For Imi/*x*Al_SBA-15 catalysts (Entry 3 vs. 4) the slightly higher epoxide conversion and the total CO_2_ consumption confirm that the sample containing the highest Al amount is more active; however, such higher catalytic activity is probably associated mainly with its significantly higher imidazolium content (*cf*. Table 1).

#### 2.2.2. CO_2_ Addition to Epichlorohydrin

In order to better understand the differences between the two imidazolium-based catalysts, additional experiments with a lower amount of catalyst and a more challenging substrate (epichlorohydrin) were performed. The previous observation of a superior activity of the imidazolium-based catalysts was further confirmed by the lack of epichlorohydrin conversion in the presence of the MgO-based systems, even with 500 mg of the more active catalyst of the series (MgO/5Al_SBA-15). On the contrary, the Imi/*x*Al_SBA-15 catalysts resulted to be active in converting epichlorohydrin (Table 3). On these two systems, recycling experiments were also performed. The catalysts recovered after the first run were less active than the fresh ones (Entry 1b vs. 1a and Entry 2b vs. 2a) and for Imi/1Al_SBA-15 a similar decrease in epichlorohydrin conversion was also observed from the second to the third run (Entry 1c vs. 1b). However, both catalysts achieved stabilization after two (Imi/5Al_SBA-15, Entry 2c vs. 2b) or three (Imi/1Al_SBA-15, Entry 1d vs. 1c) runs. Such an initial decrease in conversion from one run to the other is probably due to leaching phenomena, as also indicated by comparing the imidazolium content calculated from the chemical analysis on the fresh systems (*cf.* Table 1) and on the samples recovered after their last catalytic run (Imi/1Al_SBA-15, Entry 1d: 0.41 mmol/g; Imi/5Al_SBA-15, Entry 2c: 0.55 mmol/g). Comparing the conversion values achieved after stabilization (Entries 1c-d and 2b-c), Imi/5Al_SBA-15 is still more active than Imi/1Al_SBA-15. Interestingly, when TON (and TOF) values are calculated, virtually the same results are obtained for the two systems, indicating that the higher conversion obtained with the sample with the higher Al content is most likely due to the higher imidazolium content (*cf.* above).

Both materials can be compared with a previously reported system bearing Sn as a co-catalyst (Entry 3). For comparable conversion, selectivity, and BET surface area (282 vs. 234 m^2^/g), the Imi/5Al_SBA-15 material reported here showed TON (and TOF) 4.5 times higher.

Additional experiments were performed using the most active Imi/5Al_SBA-15 on epichlorohydrin at lower temperatures (Entries 4a–c). A less important—but not negligible—decrease in conversion was observed also at 100 °C, in agreement with a lower loss of imidazolium function during the catalytic runs (Entry 4c: 0.62 mmol/g).

Although at 100 °C Imi/5Al_SBA-15 showed a lower conversion than the previously mentioned Sn-containing catalyst (Entry 5), when TON and TOF are considered, it still results to be more active. When compared to the results obtained at 100 °C with a bromide-containing catalyst (Entry 6), Imi/5Al_SBA-15 definitely presents better performance. On the other hand, the performance of an iodide-containing material (Entry 7) is significantly better in terms of epoxide conversion and TON; however, when TOF is considered—therefore, taking into account different reaction times—Imi/5Al_SBA-15 appears even superior.

A straightforward comparison with other catalysts from the literature is often difficult owing to the different reaction conditions employed in terms of pressure, temperature, use of solvent, the addition of homogenous catalyst/co-catalyst, etc. Despite this intrinsic difficulty, in Table 3 the present catalysts are also compared with some fully heterogeneous solids from the literature. In Entries 8 and 9 results obtained at 140 °C with two metal-organic frameworks (MOFs) modified with imidazolium bromide or iodide are reported, whereas data reported in Entry 10 refer to results obtained under mild conditions on an imidazolium-containing Al-porphyrin-based ionic porous organic polymer (Al-iPOP) catalyst. In Entry 11 data on the performance of a catalyst obtained anchoring an imidazolium organic salt on Ti-SBA-15 are reported. Since reaction conditions are quite different in terms of several parameters (temperature, pressure, imidazolium content, catalyst amount, reaction time, etc.), a direct comparison with the present catalysts cannot be performed. However, although MOFs (Entries 8 and 9) contained more nucleophilic anions and were tested at a significantly higher temperature, the TON values were much lower than those obtained at 125 °C for both the present (chloride-containing) Imi/*x*Al_SBA-15 catalysts. On the other hand, the comparable TOF values obtained with the present Imi/5Al_SBA-15 catalyst at 100 °C and with the Al-iPOP system (Entry 10) at 40 °C can be related to the completely different nature of the aluminum species which are inorganic or in the form of an Al-porphyrin group, respectively. The values of TON and TOF calculated for the Ti-SBA-15-supported system (Entry 11) at 110 °C, i.e., at a somewhat intermediate temperature between those selected for the present experiments, are higher than the values obtained at 100 °C with Imi/5Al_SBA-15, but significantly lower than those determined on both the present catalysts at 125 °C.

**Table 3:**
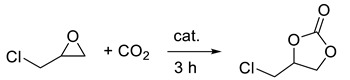
Catalytic results for epichlorohydrin conversion to the corresponding cyclic carbonate.

Entry	Catalyst	Anion	*m*_cat_ (mg)	*T* (°C)	Run ^1^	*X* (%) ^2^	*S* (%) ^2^	TON ^3^	TOF ^4^
1a	Imi/1Al_SBA-15	Cl^−^	300	125	1st	63	>95		
1b	Imi/1Al_SBA-15	Cl^−^	300	125	2nd	51	>95		
1c	Imi/1Al_SBA-15	Cl^−^	300	125	3rd	42	>95		
1d	Imi/1Al_SBA-15	Cl^−^	300	125	4th	43	>95	1073	358
2a	Imi/5Al_SBA-15	Cl^−^	300	125	1st	78	>95		
2b	Imi/5Al_SBA-15	Cl^−^	300	125	2nd	57	>95		
2c	Imi/5Al_SBA-15	Cl^−^	300	125	3rd	57	>95	1061	354
3	XS-Sn-imi [55]	Cl^−^	500	125	1st	53	>95	233	78
4a	Imi/5Al_SBA-15	Cl^−^	300	100	1st	24	>95		
4b	Imi/5Al_SBA-15	Cl^−^	300	100	2nd	20	>95		
4c	Imi/5Al_SBA-15	Cl^−^	300	100	3rd	18	>95	297	99
5	XS-Sn-imi [55]	Cl^−^	500	100	1st	40	>95	185	62
6	bV-Imi-NT-2 [60]	Br^−^	100	100	1st	9	>95	115	38
7	Imidazolium Cross-linked POSS [61]	I^−^	40	100	1st	49	>99	1371	86
8	ZnTCPP⊂(Br-)Etim-UiO-66 [62]	Br^−^	43	140	1st	87	n.d.	91	6.5
9	F-IRMOF-3-4d [63]	I^−^	300	140	1st	80	n.d.	561	374
10	Al-iPOP-2 [64]	Br^−^	3.5	40	1st	>99	>99	566	94
11	Ti-SBA-15@ILClCH_2_COO (0.5) [43]	ClCH_2_COO^−^	300	110	1st	100	99	450	150

Reaction conditions: epichlorohydrin, 24.0 mL, 307 mmol; initial CO_2_ pressure (at room temperature), 25 bar; heating rate, 1 °C/min; reaction time, 3 h; stirring speed, 500 rpm. ^1^ 1st run: fresh catalyst; 2nd run: first recycling; 3rd run: second recycling; 4th run: third recycling; when there was a loss of mass during recycling operations, the amount of epoxide was adapted to keep catalyst/epoxide ratio constant. ^2^ *X*: epoxide conversion, *S*: cyclic carbonate selectivity; determined by ^1^H NMR of the reaction mixture. ^3^ Calculated as moles of epoxides converted/moles of imidazolium sites; amount of imidazolium per mass of catalyst, determined by combustion chemical analysis considering the N percentage: Entry 1d, 0.41 mmol/g; Entry 2c, 0.55 mmol/g; Entry 4c, 0.62 mmol/g. ^4^ Calculated as TON/reaction time. Entry 7: reaction time, 16 h. Entry 8: constant CO_2_ pressure, 1 bar; reaction time, 14 h; TON calculated considering 86.9% yield and 0.95 mol % imidazolium content. Entry 9: CO_2_ pressure, 20 bar; reaction time, 1.5 h; TON calculated considering 80% conversion and 100% selectivity with 0.2 mol epichlorohydrin and 170 mg catalyst with 0.285 mmol imidazolium (21.3 wt% of iodine). Entry 10: CO_2_ pressure, 10 bar; reaction time, 6 h; TON calculated considering 99% yield and 0.00525 mmol imidazolium. Entry 11: CO_2_ pressure, 5 bar; reaction time, 3 h; TON calculated considering 99% yield and 0.220 mmol imidazolium.

#### 2.2.3. CO_2_ Addition to Styrene Oxide

The more active of the present catalysts (Imi/5Al_SBA-15) was tested with the further less reactive styrene oxide at 125 °C. The results, reported in Table 4 (Entries 1a-b), show that the material was immediately recyclable. In fact, the chemical analysis of the catalyst recovered after two runs revealed an imidazolium content of 0.74 mmol/g, i.e., virtually the same obtained on the fresh sample (*cf.* Table 1). Probably the extent of leaching phenomena depends on the nature of the epoxide reactant, being quite important with epichlorohydrin but practically absent with styrene oxide. This is consistent with previous results and certifies the robustness of the recycling method employed [55,59].

The present aluminum-containing sample showed higher TON and TOF values when compared to previously reported Sn- or Ti-based materials (Entries 2–4), studied in the same laboratory in the same conditions. However, the difference is less pronounced than that observed when epichlorohydrin was used (*cf.* Table 3).

Entries 5 and 6 in Table 4 refer to results reported for the styrene oxide conversion at 140 °C on the two already mentioned MOFs. TON and TOF values obtained with Imi/5Al_SBA-15 were significantly higher than those reported for the bromide-containing sample (Entry 5) also with this epoxide. On the contrary, the iodide-containing MOF (Entry 6) was characterized by higher TON and TOF values; however, such a difference could be related to the higher temperature and the more nucleophilic character of the anion. Actually, when other iodide-containing systems were used at the same (or very closed) temperature as the present catalyst (Entries 7 and 8), TON and TOF values were significantly lower.

From the comparison of the performance of the present Imi/*x*Al_SBA-15 systems with the results already reported in the literature (either by the present authors or by other researchers), the present aluminum-containing systems appear superior to—or at least competitive with—most of the catalysts already studied for the conversion of different epoxides to the corresponding cyclic carbonates. They are, therefore, promising materials and worthy of further investigation. In particular, it could be interesting to evaluate their performance in the conversion of the even more challenging disubstituted epoxides, such as cyclohexene oxide.

## 3. Materials and Methods

Pluronic P123 triblock copolymer (EO_20_PO_70_EO_20_), tetraethylorthosilicate (TEOS, 98%), Al(NO_3_)_3_·9H_2_O (≥98%), toluene (anhydrous, 99.8%), 3-(chloropropyl)-trimethoxysilane (≥97%), 1-methylimidazole (>99%), methanol (anhydrous, 99.8%), *n*-hexane (anhydrous, 95%), Mg(NO_3_)_2_·6H_2_O (99%), and glycidol (96%) were purchased from Sigma-Aldrich. HCl (37%) was provided by Merck. Styrene oxide and epichlorohydrin were provided by TCI.

Two aluminosilicate supports were prepared by incorporation of aluminum in the mesostructured silica SBA-15, by a modified two-step “pH-adjusting” method [67], using aluminum nitrate as the aluminum source. In a typical synthesis, 4 g of Pluronic P123 and 126 mL of a 2 M HCl aqueous solution were stirred at 40 °C until complete dissolution of the surfactant. Next, 8.5 g of TEOS was added dropwise and the solution was kept under magnetic stirring at 40 °C for 4 h. The amount of Al(NO_3_)_3_·9H_2_O required to obtain the desired Si/Al molar ratio was then added, followed by magnetic stirring for 20 h at 40 °C. The mixture was transferred into a 250 mL Teflon-lined stainless-steel autoclave and submitted to a first hydrothermal treatment at 100 °C overnight. Next, the resulting suspension was cooled to room temperature and then the pH value of the mother liquor was adjusted to 7.5 with ammonia solution under stirring. The mixture was then submitted to a second hydrothermal treatment in an autoclave at 100 °C for 48 h. Finally, the solid was separated by filtration, washed with distilled water, dried at 60 °C overnight, and calcined at 550 °C for 5 h (heating rate, 4 °C min^-1^). The aluminosilicate supports were indicated as *x*Al_SBA-15, where *x* represents the approximate nominal Al mol% content (*x* = [*n*_Al_/(*n*_Si_ + *n*_Al_)] × 100).

Two imidazolium-containing catalysts (Imi/*x*Al_SBA-15) were then obtained by functionalization of the aluminosilicate supports previously synthesized [55,56]. A one-pot procedure, consisting of reacting the *x*Al_SBA-15 supports with a mixture of 3-(chloropropyl)-trimethoxysilane and 1-methylimidazole, was employed. In a typical synthesis, 1.5 g of support (previously dried at 100 °C overnight) was dispersed in 20 mL of toluene. Then, 2.21 mL of 3-(chloropropyl)-trimethoxysilane (12 mmol) and 2 mL of 1-methylimidazole (24 mmol) were added, and the suspension was kept under reflux for 24 h. Next, the solid was filtered and washed with hot methanol (50 °C). The recovered material was then dried at 60 °C overnight and kept under reflux with methanol for 48 h to remove the imidazolium species not anchored on the surface of the support. Finally, the catalyst was filtered and dried at 100 °C overnight.

Two MgO-containing catalysts (MgO/*x*Al_SBA-15) were also prepared, by the two-solvent impregnation method on the same two *x*Al_SBA-15 supports [57,58]. For a nominal MgO content of 15 wt%, 1.2 g of support was suspended in 48 mL of *n*-hexane, used as a hydrophobic solvent, and the suspension was kept under stirring for 15 min at 400 rpm. Next, a solution prepared by dissolving ca. 7.4 mmol of Mg(NO_3_)_2_·6H_2_O in a volume of distilled water equal to the pore volume of the *x*Al_SBA-15 support, was added dropwise. The resulting dispersion was, therefore, vigorously stirred for 2 h at room temperature; then the recovered solid was dried overnight at 40 °C and finally calcined at 450 °C for 4 h.

Inductively coupled plasma optical emission spectroscopy (ICP-OES) analyses were performed with a 5110 ICP-OES spectrometer (Agilent Technologies) to determine the Al, Si, and Mg content in the supports and in the MgO-containing catalysts. Before analysis, the sample (ca. 0.015 g), pretreated at 500 °C for 12 h, was mixed with lithium tetraborate (Li_2_B_4_O_7_/sample ratio of 14–15 *w*/*w*), placed in a platinum crucible, and fused at 1000 °C in a furnace for 30 min. After cooling the melt, the resultant fusion bead was dissolved at 80 °C for about 30 min with 20 mL of HNO_3_ (0.8 M) and then diluted to a known volume by MilliQ water.

Combustion chemical analyses were performed on a Perkin-Elmer 2400 Series II analyzer. The standard was acetanilide. This instrument was mainly used for the determination of the imidazolium content through the nitrogen amount in the material.

X-ray diffraction (XRD) analyses were performed using an X3000 diffractometer (Seifert), with a *θ*-*θ* Bragg–Brentano geometry, Cu-Kα wavelength, and a graphite monochromator before the scintillation detector. The pore mesostructure of supports and catalysts was investigated by low-angle (LA) X-ray diffraction analysis in the 2*θ* range 0.8–2.4°. Structural characteristics of the MgO/*x*Al_SBA-15 catalysts were investigated in the wide-angle (WA) 2*θ* range 20–70°.

Textural analyses were carried out with an ASAP 2020 system (Micromeritics), by determining the nitrogen adsorption-desorption isotherms at −196 °C. Before analysis, the sample was heated overnight under a vacuum up to 250 °C (heating rate, 1 °C min^−1^). The specific surface area was calculated by the BET equation. The pore size distribution was determined by applying the BJH method to the isotherm desorption branch.

Solid-state NMR spectroscopy was used for characterizing supports and Imi-containing catalysts. ^27^Al direct excitation magic-angle spinning (MAS) NMR spectra of the *x*Al_SBA-15 supports were recorded at room temperature by using a Varian VNMRS 400 spectrometer operating at 9.4 T (104.2 MHz for ^27^Al) equipped with a 4 mm CP-MAS Varian/Chemagnetics probe. The sample was packed in a 4 mm zirconia rotor and spun at a spinning frequency of 12000 Hz. Measurements were performed by using a 1 s relaxation delay. The processing comprised zero-filling, Fourier transform, phase and baseline corrections. The chemical shift scale was calibrated at RT with respect to a solid sample of AlCl_3_ hexahydrate (*δ* = 0.0 ppm). Quantitative information on the ^27^Al atom environment was obtained by fitting the signals with Gaussian functions, using the software package Origin 9 from OriginLab Corporation (Northampton, MA, USA).

^29^Si and ^13^C NMR spectra were recorded on a Bruker Avance-500 spectrometer operating at 11.7T (99.3 MHz for ^29^Si and 125.7 MHz for ^13^C) using a 4 mm CP-MAS Bruker probe. The sample was packed in a zirconia rotor and measured with spinning frequencies of 8000 Hz. Cross-polarization CP-MAS spectra were recorded using a 5 s relaxation delay and 5 ms contact time for ^29^Si and 2 ms for ^13^C. The processing comprised exponential multiplication of the FID with a line broadening factor of 30 Hz for ^29^Si or 10 Hz for ^13^C, zerofilling, Fourier transform, phase and baseline corrections. The chemical shift scale was calibrated at room temperature with respect to a sample of solid 3-(trimethylsilyl)-1-propanesulfonic acid sodium salt (DSS) (0.0 ppm) purchased from Sigma-Aldrich (St. Louis, MO, USA). 

The acidic surface properties of the supports were investigated by adsorption microcalorimetry, using ammonia as the probe gas. A Tian–Calvet heat-flow microcalorimeter (Setaram) equipped with a volumetric vacuum line was used for microcalorimetric measurements. Each sample (0.1 g, 40–80 mesh) was pretreated at 250 °C for 12 h under vacuum (5 × 10^−5^ mbar). Adsorption was carried out at 80 °C by admitting successive doses of the probe gas and recording the thermal effect. The equilibrium pressure relative to each adsorbed amount was measured by means of a differential pressure gauge (Datametrics) and the thermal effect was recorded. The run was stopped at a final equilibrium pressure of about 1.3 mbar. The adsorption temperature was maintained at 80 °C, in order to limit physisorption. The adsorption isotherm (relating the amount of probe gas adsorbed with the corresponding equilibrium pressure) and the calorimetric isotherm (relating the integral heat of adsorption with the corresponding equilibrium pressure) were obtained from each adsorption run. Combining the two sets of data, a plot of the differential heat of adsorption as a function of the adsorbed amount was drawn, which gives information on the influence of the surface coverage on the energetics of the adsorption.

All catalytic tests were performed in a Cambridge Design Bullfrog batch reactor. All catalysts were dried overnight at 100 °C prior to the catalytic test. In a typical test, the solid was weighted in a Teflon vial. The epoxide (24.0 mL) was added without solvent. Then, the reactor was closed, purged with a gentle flow of N_2_ for 10 min and pressurized with CO_2_ (25 bar) at room temperature. The mixture was stirred at 500 rpm using a PTFE-coated mechanical stirrer and heated to the desired reaction temperature with a controlled ramp. During the test, both temperature and pressure were monitored and recorded. At the end of the reaction, the reactor was cooled down to room temperature, the pressure was released and the reactor opened. The reaction mixture was submitted to centrifugation for 10 min at 4500 rpm and the supernatant was analyzed by ^1^H-NMR using DMSO-d6 as solvent. The experimental error, obtained by repeating some tests, was found to be always within 5%.

The catalysts were submitted to washing prior to reuse. After removal of the epoxide/carbonate mixture by centrifugation, the catalyst was dispersed in toluene, submitted to sonication for 15 min and centrifuged. The supernatant was removed. The operation was repeated with ethanol as solvent.

## 4. Conclusions

Two mesostructured aluminosilicates with a nominal Al content of 1 and 5 mol% were synthesized. The aluminum loading was quantified by ICP-OES analysis and its incorporation in the silica framework was evaluated by ^27^Al MAS NMR. The acidity of the material was evaluated via NH_3_ adsorption microcalorimetry.

Thereafter, two strategies of post-functionalization were employed. The first strategy led to a fully inorganic material decorated with MgO nanoparticles. The second procedure allowed obtaining an organic-inorganic hybrid solid functionalized with imidazolium chloride. The efficiency of both syntheses was proved by ICP-OES, combustion chemical analysis, ^29^Si and ^13^C MAS NMR spectroscopy.

All the materials were used as catalysts for the conversion of glycidol to the corresponding cyclic carbonate. MgO-containing catalysts showed moderate activity compared to the imidazolium chloride-based systems. This result is attributed to the good nucleophilic features of the chloride anion compared to the MgO which can be considered a Lewis base. The most active imidazolium-containing materials were tested over epichlorohydrin and styrene oxide as substrates. In all cases, the system with the higher Al loading showed the best activities, out-competing (in terms of TON) most of the catalysts already reported in the literature.

## Figures and Tables

**Table 1:** Compositional and textural properties of supports and catalysts.

Sample	Al/(Al + Si) (mol%) ^1^	Al_framework_ (mol%) ^2^	Imidazolium Content (mmol/g) ^3^	MgO Content (mmol/g) ^1^	*S*_BET_ (m^2^/g)	*V*_p_ (cm^3^/g)
1Al_SBA-15	1.13	17	-	-	554	0.96
5Al_SBA-15	4.02	25	-	-	409	0.94
MgO/1Al_SBA-15	1.11	-	-	4.05	235	0.60
MgO/5Al_SBA-15	3.94	-	-	4.14	240	0.61
Imi/1Al_SBA-15	-	-	0.55	-	349	0.81
Imi/5Al_SBA-15	-	-	0.75	-	282	0.70

^1^ Determined by ICP-OES analysis. ^2^ Determined by ^27^Al MAS NMR. ^3^ Determined by combustion chemical analysis considering the nitrogen percentage.

**Table 2:**
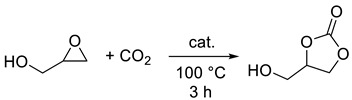
Catalytic results for glycidol conversion to the corresponding cyclic carbonate.

Entry	Catalyst	*X* (%) ^1^	*S* (%) ^1^
1	MgO/1Al_SBA-15	23	>95
2	MgO/5Al_SBA-15	32	>95
3	Imi/1Al_SBA-15	86	>95
4	Imi/5Al_SBA-15	89	>95

Reaction conditions: glycidol, 24.0 mL, 362 mmol; catalyst, 500 mg; initial CO_2_ pressure (at room temperature), 25 bar; heating rate, 1 °C/min; reaction temperature, 100 °C; reaction time, 3 h; stirring speed, 500 rpm. ^1^ *X*: epoxide conversion, *S*: cyclic carbonate selectivity; determined by ^1^H NMR of the reaction mixture.

**Table 4:**
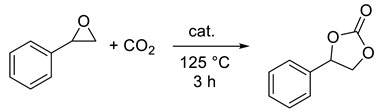
Catalytic results for styrene oxide conversion to the corresponding cyclic carbonate.

Entry	Catalyst	Anion	*m*_cat_ (mg)	*T* (°C)	Run ^1^	*X* (%) ^2^	*S* (%) ^2^	TON ^3^	TOF ^4^
1a	Imi/5Al_SBA-15	Cl^−^	300	125	1st	18	>95		
1b	Imi/5Al_SBA-15	Cl^−^	300	125	2nd	17	>95	162	54
2	XS-Sn-imi [55]	Cl^−^	500	125	1st	32	>95	96	32
3	Ti-SiO_2_-E-imi [56]	Cl^−^	500	125	1st	42	>95	127	42
4	Sn-A-imi(II)-Cl [59]	Cl^−^	500	125	1st	22	>95	108	36
5	ZnTCPP⊂(Br-)Etim-UiO-66 [62]	Br^−^	43	140	1st	53	n.d.	56	4.0
6	F-IRMOF-3-4d [63]	I^−^	170	140	1st	84	n.d.	589	118
7	SiO_2_-p-xylene-I [65]	I^−^	100	125	1st	37	>99	88	29
8	(I-)Meim-UiO-66 [66]	I^−^	50	120	1st	46	71	63	2.6

Reaction conditions: styrene oxide, 24.0 mL, 211 mmol; catalyst, 300 mg; initial CO_2_ pressure (at room temperature), 25 bar; heating rate, 1 °C/min; reaction time, 3 h; stirring speed, 500 rpm. ^1^ 1st run: fresh catalyst; 2nd run: first recycling; when there was a loss of mass during recycling operations, the amount of epoxide was adapted to keep catalyst/epoxide ratio constant. ^2^ *X*: epoxide conversion, *S*: cyclic carbonate selectivity; determined by ^1^H NMR of the reaction mixture. ^3^ Calculated as moles of epoxides converted/moles of imidazolium sites; amount of imidazolium per mass of catalyst, determined by combustion chemical analysis considering the N percentage: Entry 1b, 0.74 mmol/g. ^4^ Calculated as TON/reaction time. Entry 5: constant CO_2_ pressure, 1 bar; reaction time, 14 h; TON calculated considering 52.8% yield and 0.95 mol % imidazolium content. Entry 6: CO_2_ pressure, 20 bar; reaction time, 5 h; TON calculated considering 84% conversion and 100% selectivity with 200 mmol styrene oxide and 0.285 mmol imidazolium (21.3 wt% of iodine). Entry 7: styrene oxide, 46.7 mmol; mesitylene, 4.67 mmol; CO_2_ pressure, 80 bar; reaction time, 3 h. Entry 8: TON calculated considering 10 mmol styrene oxide and 0.052 mmol imidazolium.

## Data Availability

Not applicable.

## Supplementary Materials

The following supporting information can be downloaded at: https://www.mdpi.com/article/10.3390/molecules27248883/s1, Figure S1: Pore size distribution for the *x*Al_SBA-15 supports and the Imi/*x*Al_SBA-15 and MgO/*x*Al_SBA-15 catalysts; Figure S2: Wide-angle XRD patterns of MgO/1Al_SBA-15 (a) and MgO/5Al_SBA-15 (b).

## Sample Availability

Samples of the compounds are available from the authors.

## References

[1] North M., Pasquale R., Young C. (2010). Synthesis of cyclic carbonates from epoxides and CO_2_. Green Chem..

[2] Aresta M. (2010). Carbon Dioxide as Chemical Feedstock.

[3] Guo L., Lamb K.J., North K. (2021). Recent developments in organocatalysed transformations of epoxides and carbon dioxide into cyclic carbonates. Green Chem..

[4] Styring P., Quadrelli E.A., Armstrong K. (2014). Carbon Dioxide Utilisation: Closing the Carbon Cycle.

[5] Kim H.S., Bae J.Y., Lee J.S., Kwon O.-S., Jelliarko P., Lee S.D., Lee S.H. (2005). Phosphine-bound zinc halide complexes for the coupling reaction of ethylene oxide and carbon dioxide. J. Catal..

[6] Sun J., Wang L., Zhang S., Li Z., Zhang X., Dai W., Mori R. (2006). ZnCl_2_/phosphonium halide: An efficient Lewis acid/base catalyst for the synthesis of cyclic carbonate. J. Mol. Catal. A Chem..

[7] Barbarini A., Maggi R., Mazzacani A., Mori G., Sartori G., Sartorio R. (2003). Cycloaddition of CO_2_ to epoxides over both homogeneous and silica-supported guanidine catalysts. Tetrahedron Lett..

[8] Jiang J.-L., Hua R. (2006). Efficient DMF-Catalyzed Coupling of Epoxides with CO_2_ under Solvent-Free Conditions to Afford Cyclic Carbonates. Synth. Commun..

[9] Paddock R.L., Hiyama Y., McKay J.M., Nguyen S.B.T. (2004). Co(III) porphyrin/DMAP: An efficient catalyst system for the synthesis of cyclic carbonates from CO_2_ and epoxides. Tetrahedron Lett..

[10] Srivastava R., Bennur T.H., Srinivas D. (2005). Factors affecting activation and utilization of carbon dioxide in cyclic carbonates synthesis over Cu and Mn peraza macrocyclic complexes. J. Mol. Catal. A Chem..

[11] Bu Z., Qin G., Cao S. (2007). A ruthenium complex exhibiting high catalytic efficiency for the formation of propylene carbonate from carbon dioxide. J. Mol. Catal. A Chem..

[12] Calo V., Nacci A., Monopoli A., Fanizzi A. (2002). Cyclic carbonate formation from carbon dioxide and oxiranes in tetrabutylammonium halides as solvents and catalysts. Org. Lett..

[13] Sun J., Fujita S.-I., Arai M. (2005). Development in the green synthesis of cyclic carbonate from carbon dioxide using ionic liquids. J. Organomet. Chem..

[14] Zhou Y., Hu S., Ma X., Liang S., Jiang T., Han B. (2008). Synthesis of cyclic carbonates from carbon dioxide and epoxides over betaine-based catalysts. J. Mol. Catal. A Chem..

[15] Sun J., Ren J., Zhang S., Cheng W. (2009). Water as an efficient medium for the synthesis of cyclic carbonate. Tetrahedron Lett..

[16] Jutz F., Andanson J.M., Baiker A. (2011). Ionic liquids and dense carbon dioxide: A beneficial biphasic system for catalysis. Chem. Rev..

[17] Wang J.-Q., Cheng W.-G., Sun J., Shi T.-Y., Zhang X.-P., Zhang S.-J. (2014). Efficient fixation of CO_2_ into organic carbonates catalyzed by 2-hydroxymethyl-functionalized ionic liquids. RSC Adv..

[18] Dai W.-L., Luo S.-L., Yin S.-F., Au C.-T. (2009). The direct transformation of carbon dioxide to organic carbonates over heterogeneous catalysts. Appl. Catal. A Gen..

[19] Wong W.L., Chan P.H., Zhou Z.Y., Lee K.H., Cheung K.C., Wong K.Y. (2008). A robust ionic liquid as reaction medium and efficient organocatalyst for carbon dioxide fixation. ChemSusChem.

[20] Kawanami H., Sasaki A., Matsui K., Ikushima Y. (2003). A rapid and effective synthesis of propylene carbonate using a supercritical CO_2_-ionic liquid system. Chem. Commun..

[21] Bobbink F.D., Dyson P.J. (2016). Synthesis of carbonates and related compounds incorporating CO_2_ using ionic liquid-type catalysts: State-of-the-art and beyond. J. Catal..

[22] North M., Pasquale R. (2009). Mechanism of cyclic carbonate synthesis from epoxides and CO_2_. Angew. Chem. Int. Ed..

[23] Pescarmona P.P., Taherimehr M. (2012). Challenges in the catalytic synthesis of cyclic and polymeric carbonates from epoxides and CO_2_. Catal. Sci. Technol..

[24] Buaki-Sogo M., Garcia H., Aprile C. (2015). Imidazolium-based silica microreactors for the efficient conversion of carbon dioxide. Catal. Sci. Technol..

[25] Xie Y., Zhang Z., Jiang T., He J., Han B., Wu T., Ding K. (2007). CO_2_ cycloaddition reactions catalyzed by an ionic liquid grafted onto a highly cross-linked polymer matrix. Angew. Chem. Int. Ed..

[26] Dai W.-L., Chen L., Yin S.-F., Li W.-H., Zhang Y.-Y., Luo S.-L., Au C.-T. (2010). High-Efficiency Synthesis of Cyclic Carbonates from Epoxides and CO_2_ over Hydroxyl Ionic Liquid Catalyst Grafted onto Cross-Linked Polymer. Catal. Lett..

[27] Watile R.A., Deshmukh K.M., Dhake K.P., Bhanage B.M. (2012). Efficient synthesis of cyclic carbonate from carbon dioxide using polymer anchored diol functionalized ionic liquids as a highly active heterogeneous catalyst. Catal. Sci. Technol..

[28] Shi T.-Y., Wang J.-Q., Sun J., Wang M.-H., Cheng W.-G., Zhang S.-J. (2013). Efficient fixation of CO_2_ into cyclic carbonates catalyzed by hydroxyl-functionalized poly(ionic liquids). RSC Adv..

[29] Sun J., Wang J., Cheng W., Zhang J., Li X., Zhang S., She Y. (2012). Chitosan functionalized ionic liquid as a recyclable biopolymer-supported catalyst for cycloaddition of CO_2_. Green Chem..

[30] Roshan K.R., Mathai G., Kim J., Tharun J., Park G.-A., Park D.-W. (2012). A biopolymer mediated efficient synthesis of cyclic carbonates from epoxides and carbon dioxide. Green Chem..

[31] Xiao L.-F., Li F.-W., Peng J.-J., Xia C.-G. (2006). Immobilized ionic liquid/zinc chloride: Heterogeneous catalyst for synthesis of cyclic carbonates from carbon dioxide and epoxides. J. Mol. Catal. A Chem..

[32] Dai W.-L., Chen L., Yin S.-F., Luo S.-L., Au C.-T. (2010). 3-(2-Hydroxyl-Ethyl)-1-Propylimidazolium Bromide Immobilized on SBA-15 as Efficient Catalyst for the Synthesis of Cyclic Carbonates via the Coupling of Carbon Dioxide with Epoxides. Catal. Lett..

[33] Srivastava R., Srinivas D., Ratnasamy P. (2005). CO_2_ activation and synthesis of cyclic carbonates and alkyl/aryl carbamates over adenine-modified Ti-SBA-15 solid catalysts. J. Catal..

[34] Srivastava R., Srinivas D., Ratnasamy P. (2006). Sites for CO_2_ activation over amine-functionalized mesoporous Ti(Al)-SBA-15 catalysts. Micropor. Mesopor. Mater..

[35] Srinivas D., Ratnasamy P. (2007). Spectroscopic and catalytic properties of SBA-15 molecular sieves functionalized with acidic and basic moieties. Micropor. Mesopor. Mater..

[36] Baleizão C., Gigante B., Sabater M.J., Garcia H., Corma A. (2002). On the activity of chiral chromium salen complexes covalently bound to solid silicates for the enantioselective epoxide ring opening. App. Catal. A Gen..

[37] Alvaro M., Baleizao C., Das D., Carbonell E., García H. (2004). CO_2_ fixation using recoverable chromium salen catalysts: Use of ionic liquids as cosolvent or high-surface-area silicates as supports. J. Catal..

[38] Ramin M., Jutz F., Grunwaldt J.-D., Baiker A. (2005). Solventless synthesis of propylene carbonate catalysed by chromium–salen complexes: Bridging homogeneous and heterogeneous catalysis. J. Mol. Catal. A Chem..

[39] Bhanage B.M., Fujita S.-I., Ikushima Y., Arai M. (2001). Synthesis of dimethyl carbonate and glycols from carbon dioxide, epoxides, and methanol using heterogeneous basic metal oxide catalysts with high activity and selectivity. Appl. Catal. A Gen..

[40] Yamaguchi K., Ebitani K., Yoshida T., Yoshida H., Kaneda K. (1999). Mg-Al Mixed Oxides as Highly Active Acid-Base Catalysts for Cycloaddition of Carbon Dioxide to Epoxides. J. Am. Chem. Soc..

[41] Doskocil E.J. (2004). Ion-exchanged ETS-10 catalysts for the cycloaddition of carbon dioxide to propylene oxide. Micropor. Mesopor. Mater..

[42] Doskocil E.J. (2005). Effect of water and alkali modifications on ETS-10 for the cycloaddition of CO_2_ to propylene oxide. J. Phys. Chem. B.

[43] Hu Y.L., Wang H.B., Chen Z.W., Li X.G. (2018). Titanium Incorporated Mesoporous Silica Immobilized Functional Ionic Liquid as an Efficient Reusable Catalyst for Cycloaddition of Carbon Dioxide to Epoxides. ChemistrySelect.

[44] Borade R.B., Clearfield A. (1995). Synthesis of aluminum rich MCM-41. Catal. Lett..

[45] Kloetstra K.R., Zandbergen H.W., van Bekkum H. (1995). MCM-41 type materials with low Si/Al ratios. Catal. Lett..

[46] Chen J., Chen T., Guan N., Wang J. (2004). Dealumination process of zeolite omega monitored by ^27^Al 3QMAS NMR spectroscopy. Catal. Today.

[47] Li S., Zheng A., Su Y., Fang H., Shen W., Yu Z., Chen L., Deng F. (2010). Extra-framework aluminium species in hydrated faujasite zeolite as investigated by two-dimensional solid-state NMR spectroscopy and theoretical calculations. Phys. Chem. Chem. Phys..

[48] Meloni D., Perra D., Monaci R., Cutrufello M.G., Rombi E., Ferino I. (2016). Transesterification of Jatropha curcas oil and soybean oil on Al-SBA-15 catalysts. Appl. Catal. B Environ..

[49] Colón G., Ferino I., Rombi E., Selli E., Forni L., Magnoux P., Guisnet M. (1998). Liquid-phase alkylation of naphthalene by isopropanol over zeolites. Part 1: HY zeolites. Appl. Catal. A Gen..

[50] Thommes M., Kaneko K., Neimark A.V., Olivier J.P., Rodriguez-Reinoso F., Rouquerol J., Sing K.S.W. (2015). Physisorption of gases, with special reference to the evaluation of surface area and pore size distribution (IUPAC Technical Report). Pure Appl. Chem..

[51] Zhao D., Feng J., Huo Q., Melosh N., Fredrickson G.H., Chmelka B.F., Stucky G.D. (1998). Triblock copolymer syntheses of mesoporous silica with periodic 50 to 300 angstrom pores. Science.

[52] Rombi E., Cutrufello M.G., Cannas C., Occhiuzzi M., Onida B., Ferino I. (2012). Gold-assisted E′ centres formation on the silica surface of Au/SBA-15 catalysts for low temperature CO oxidation. Phys. Chem. Chem. Phys..

[53] Varghese S., Cutrufello M.G., Rombi E., Monaci R., Cannas C., Ferino I. (2014). Mesoporous hard-templated Me–Co [Me = Cu, Fe] spinel oxides for water gas shift reaction. J. Porous Mater..

[54] Atzori L., Cutrufello M.G., Meloni D., Monaci R., Cannas C., Gazzoli D., Sini M.F., Deiana P., Rombi E. (2017). CO_2_ methanation on hard-templated NiO-CeO_2_ mixed oxides. Int. J. Hydrogen Energy.

[55] Comès A., Collard X., Fusaro L., Atzori L., Cutrufello M.G., Aprile C. (2018). Bi-functional heterogeneous catalysts for carbon dioxide conversion: Enhanced performances at low temperature. RSC Adv..

[56] Comès A., Poncelet R., Pescarmona P.P., Aprile C. (2021). Imidazolium-based titanosilicate nanospheres as active catalysts in carbon dioxide conversion: Towards a cascade reaction from alkenes to cyclic carbonates. J. CO2 Util..

[57] Imperor-Clerc M., Bazin D., Appay M.-D., Beaunier P., Davidson A. (2004). Crystallization of β-MnO_2_ Nanowires in the Pores of SBA-15 Silicas: In Situ Investigation Using Synchrotron Radiation. Chem. Mater..

[58] Mureddu M., Ferino I., Rombi E., Cutrufello M.G., Deiana P., Ardu A., Musinu A., Piccaluga G., Cannas C. (2012). ZnO/SBA-15 composites for mid-temperature removal of H_2_S: Synthesis, performance and regeneration studies. Fuel.

[59] Comès A., Fiorilli S., Aprile C. (2020). Multifunctional heterogeneous catalysts highly performing in the conversion of carbon dioxide: Mechanistic insights. J. CO2 Util..

[60] Buaki-Sogó M., Vivian A., Bivona L.A., García H., Gruttadauria M., Aprile C. (2016). Imidazolium functionalized carbon nanotubes for the synthesis of cyclic carbonates: Reducing the gap between homogeneous and heterogeneous catalysis. Catal. Sci. Technol..

[61] Calabrese C., Fusaro L., Liotta L.F., Giacalone F., Comès A., Campisciano V., Aprile C., Gruttadauria M. (2019). Efficient Conversion of Carbon Dioxide by Imidazolium-Based Cross-Linked Nanostructures Containing Polyhedral Oligomeric Silsesquioxane (POSS) Building Blocks. ChemPlusChem.

[62] Liang J., Xie Y.-Q., Wu Q., Wang X.-Y., Liu T.-T., Li H.-F., Huang Y.-B., Cao R. (2018). Zinc Porphyrin/Imidazolium Integrated Multivariate Zirconium Metal-Organic Frameworks for Transformation of CO_2_ into Cyclic Carbonates. Inorg. Chem..

[63] Zhou X., Zhang Y., Yang X., Zhao L., Wang G. (2012). Functionalized IRMOF-3 as efficient heterogeneous catalyst for the synthesis of cyclic carbonates. J. Mol. Catal. A Chem..

[64] Chen Y., Luo R., Xu Q., Jiang J., Zhou X., Ji H. (2017). Charged Metalloporphyrin Polymers for Cooperative Synthesis of Cyclic Carbonates from CO_2_ under Ambient Conditions. ChemSusChem.

[65] Agrigento P., Al-Amsyar S.M., Sorée B., Taherimehr M., Gruttadauria M., Aprile C., Pescarmona P.P. (2014). Synthesis and high-throughput testing of multilayered supported ionic liquid catalysts for the conversion of CO_2_ and epoxides into cyclic carbonates. Catal. Sci. Technol..

[66] Liang J., Chen R.-P., Wang X.-Y., Liu T.-T., Wang X.-S., Huang Y.-B., Cao R. (2017). Postsynthetic ionization of an imidazole-containing metal-organic framework for the cycloaddition of carbon dioxide and epoxides. Chem. Sci..

[67] Ungureanu A., Dragoi B., Hulea V., Cacciaguerra T., Meloni D., Solinas V., Dumitriu E. (2012). Effect of aluminium incorporation by the “pH-adjusting” method on the structural, acidic and catalytic properties of mesoporous SBA-15. Micropor. Mesopor. Mater..

