# Supplementary material for: Aluminosilicate-Supported Catalysts for the Synthesis of Cyclic Carbonates by Reaction of CO2 with the Corresponding Epoxides
Source: Molecules. 2022 Dec 14;27(24):8883. doi: 10.3390/molecules27248883 (PMC9782148; doi:10.3390/molecules27248883)
Supplement: Supplementary file 1 [file molecules-27-08883-s001.zip › molecules-2029542-SI.pdf]

Aluminosilicate-Supported Catalysts for the Synthesis of Cyclic Carbonates by Reaction of CO_2 with the Corresponding Epoxides

Luciano Atzori [1,†], Adrien Comès [2,†], Luca Fusaro [2], Carmela Aprile [2,*] and Maria Giorgia Cutrufello [1,3,*]

1 Dipartimento di Scienze Chimiche e Geologiche, Università di Cagliari, 09042 Monserrato, Italy; lucianoatzori@hotmail.it
2 Unit of Nanomaterial Chemistry, Department of Chemistry, University of Namur, 5000 Namur, Belgium
3 Consorzio Interuniversitario Nazionale per la Scienza e Tecnologia dei Materiali (INSTM), Unità di Cagliari, 50121 Firenze, Italy
* Correspondence: carmela.aprile@unamur.be (C.A.); gcutrufe@unica.it (M.G.C.)
† These authors contributed equally to this work.

Supplementary Materials

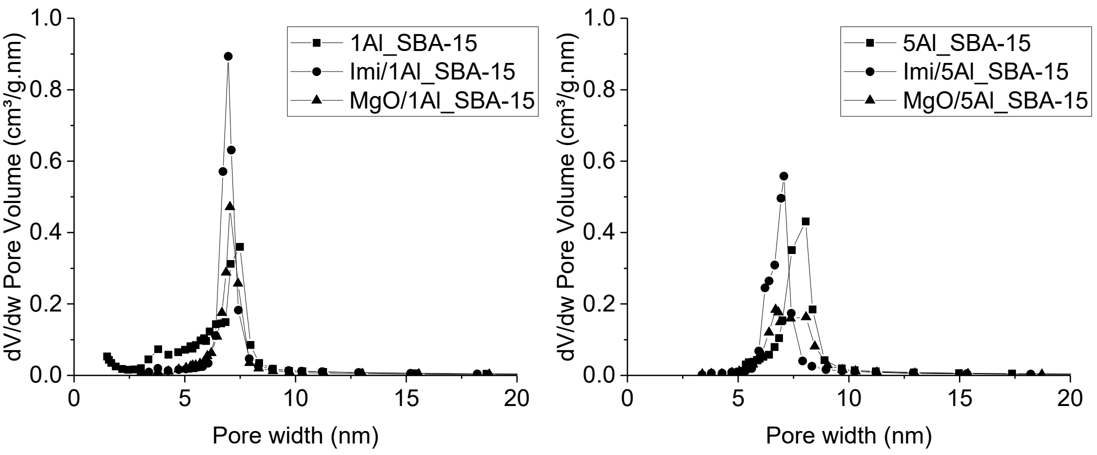

Figure S1. Pore size distribution for the *x*Al_SBA-15 supports and the Imi/*x*Al_SBA-15 and MgO/*x*Al_SBA-15 catalysts.

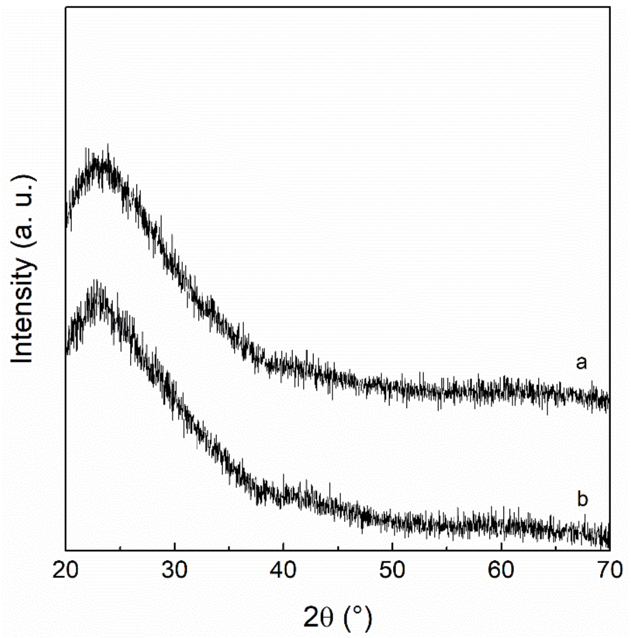

Figure S2. Wide-angle XRD patterns of MgO/1Al_SBA-15 (a) and MgO/5Al_SBA-15 (b).
